# Activity of a vmPFC-DRN Pathway Corresponds With Resistance to Acute Social Defeat Stress

**DOI:** 10.3389/fncir.2020.00050

**Published:** 2020-10-16

**Authors:** J. Alex Grizzell, Thomas T. Clarity, Nate B. Graham, Brooke N. Dulka, Matthew A. Cooper

**Affiliations:** ^1^Department of Psychology, NeuroNET Research Center, The University of Tennessee, Knoxville, Knoxville, TN, United States; ^2^Department of Psychology and Neuroscience, Center for Neuroscience Research, University of Colorado Boulder, Boulder, CO, United States

**Keywords:** acute stress, resilience, prefrontal cortex, raphe, social defeat, dominance

## Abstract

The ventromedial prefrontal cortex (vmPFC) plays a critical role in stress resilience through top-down inhibition of key stress-sensitive limbic and hindbrain structures, including the dorsal raphe nucleus (DRN). In a model of experience-dependent stress resistance, socially dominant Syrian hamsters display fewer signs of anxiety following acute social defeat when compared to subordinate or control counterparts. Further, dominants activate vmPFC neurons to a greater degree during stress than do subordinates and become stress-vulnerable following pharmacological inhibition of the vmPFC. Dominants also display fewer stress-activated DRN neurons than subordinates do, suggesting that dominance experience gates activation of vmPFC neurons that inhibit the DRN during social defeat stress. To test whether social dominance alters stress-induced activity of a vmPFC-DRN pathway, we injected a retrograde tracer, cholera toxin B (CTB), into the DRN of dominant, subordinate, and control hamsters and used a dual-label immunohistochemical approach to identify vmPFC neurons co-labeled with CTB and the defeat-induced expression of an immediate early gene, cFos. Results indicate that dominant hamsters display more cFos+ and dual-labeled cells in layers V/VI of infralimbic and prelimbic subregions of the vmPFC compared to other animals. Furthermore, vmPFC-DRN activation corresponded directly with proactive behavioral strategies during defeat, which is indicative of stress resilience. Together, results suggest that recruiting the vmPFC-DRN pathway during acute stress corresponds with resistance to the effects of social defeat in dominant hamsters. Overall, these findings indicate that a monosynaptic vmPFC-DRN pathway can be engaged in an experience-dependent manner, which has implications for behavioral interventions aimed at alleviating stress-related psychopathologies.

## Introduction

While exposure to traumatic psychological stress is a key risk factor for the development of various psychopathologies such as posttraumatic stress disorder (PTSD) and major depressive disorder (MDD), resilience to stress appears to be the rule rather than the exception. For example, of the 9 in 10 individuals estimated to encounter a traumatic stressor during their lifetime, only about 1 in 10 develop PTSD (Southwick and Charney, 2012). Accumulating evidence confirms that stress resilience can be learned, thereby implicating experience-dependent changes within the brain, which might be therapeutically recruited to promote resistance to stress-related impairments. This realization has encouraged numerous research efforts focused on elucidating the relationship between brain regions that underlie individual differences in stress processing, for example the ventromedial aspect of the prefrontal cortex (vmPFC) and the serotonin-producing, dorsal raphe nuclei (DRN).

During acutely distressful experiences, greater vmPFC engagement is seen in stress-resilient humans (Sinha et al., 2016) and non-human animals (Baratta et al., 2009; Morrison et al., 2014), whereas reduced activation corresponds with vulnerability to stress-induced impairments. Similarly, several acutely administered traumatic stressors stimulate serotonergic activity within DRN, though in an opposing manner with activation correlating positively with stress susceptibility (Amat et al., 2005; Paul et al., 2011; Cooper et al., 2017). Taken together, these findings indicate that an inverse relationship between vmPFC and DRN activity coincides with resistance to the adverse effects of acute traumatic stress.

The DRN receives substantial glutamatergic projections from the vmPFC, which preferentially synapse onto inhibitory GABAergic microcircuits (Jankowski and Sesack, 2004; Challis et al., 2014). Recruitment of this pathway during trauma is thought to promote resistance by suppressing stress-induced serotonergic activity. For instance, in a model of learned controllability, rats who exert control over the duration of a tail shock more readily recruit vmPFC-DRN projections when compared with those not having learned control (Baratta et al., 2009, 2019). Importantly, learning controllability affords resistance to the effects of tail-shock stress seen in those without control, such as learned helplessness, downward mobility of social dominance, decreased aggression, anhedonia, neophobia, and various impairments of fear learning (Maier and Seligman, 2016). Furthermore, learning control produces resilience toward future stressors, even if controllability is denied (Amat et al., 2008). In another animal model, optogenetic stimulation of the vmPFC-DRN circuit during an acute forced swim stressor promotes proactive behavioral responses (Warden et al., 2012), which have been argued to index stress resilience (e.g., Wood et al., 2010). In our laboratory, we have shown that male Syrian hamsters who maintain social dominance for 2 weeks exhibit fewer negative behavioral (Morrison et al., 2014), neuroendocrine (Dulka et al., 2018b), and neurochemical (Dulka et al., 2017) consequences of social defeat stress compared to subordinates or controls. Importantly, such status-dependent changes in behavior require recruitment of vmPFC neurons (Morrison et al., 2013) and correspond with reduced activity of the DRN during social and non-social acute stress (Cooper et al., 2017). Moreover, pharmacologically reducing DRN activity during defeat eliminates the characteristic social anxiety that follows (Cooper et al., 2008). That said, it is unknown whether the relationship between enhanced vmPFC activity and reduced DRN activity in dominant hamsters is merely coincidental, indirect, or instead due to top-down control via a monosynaptic vmPFC-DRN circuit activated during stress.

In this study, we tested the hypothesis that recruitment of a monosynaptic vmPFC-DRN circuit corresponds with stress resilience. More specifically, we predicted that stress-resistant dominant Syrian hamsters recruit more DRN-projecting neurons in the vmPFC than subordinate or control counterparts during acute social defeat stress. To address this question, we used a retrograde-tracing approach to identify vmPFC neurons that project to the DRN and co-labeled the immediate early gene (IEG), cFos, to index neural activity following acute social defeat stress.

## Materials and Methods

### Subjects

Male Syrian hamsters, *Mesocricetus auratus*, bred in-house from progenitors obtained from Charles River Laboratories, were group housed until adulthood (9 weeks old, 120–180 g). All animals were housed in a humidity- and temperature-controlled colony room (21 ± 2°C) on a 14:10-h light:dark cycle in polycarbonate cages (12 cm × 27 cm × 16 cm) with corncob bedding, cotton nesting materials, and wire mesh tops with food and water available *ad libitum*. All behavioral procedures were performed during the first 3 h of the dark phase, were approved by the University of Tennessee Institutional Animal Care and Use Committee, and were conducted in accordance with the National Institutes of Health Guide for the Care and Use of Laboratory Animals. Our final sample size across groups was 51 animals; excluded animals and criteria are noted below.

### Formation and Maintenance of Dominance Relationships

**FIGURE 1:**
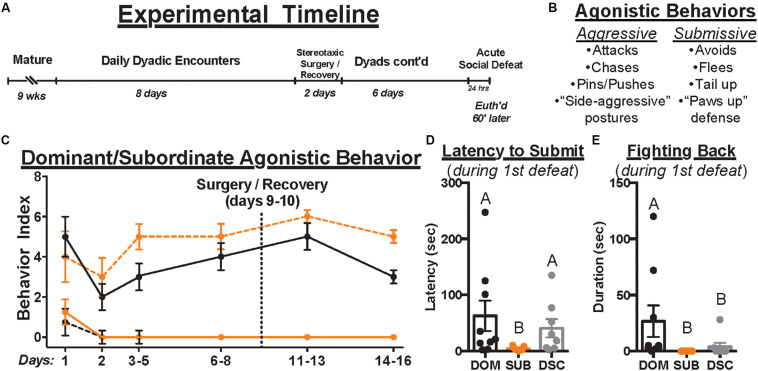
The experimental timeline is depicted in **(A)**. Upon maturation, male Syrian hamsters were assigned to a dyad with a weight- and age-matched conspecific and paired 5 min daily for a total of 14 days. Dyads were paused on days 9–10 to permit stereotaxic injection of retrograde tracer, CTB, directly into the DRN. Control hamsters remained group housed until surgery but were individually housed immediately afterward and until euthanasia. After surgical recovery, daily dyadic encounters resumed on days 11–16 where applicable. On day 17, all animals received acute social defeat or no-stress control procedures followed 60 min later by euthanasia to time-lock cFos expression within stress-activated neurons (i.e., 90 min following the start of defeat episodes). During dominance and defeat encounters, aggressive and submissive behaviors were scored per the ethogram summarized in **(B)**. The occurrence of each behavior was recorded daily to index dominance status over time **(C)**. Black and orange lines indicate summated behaviors of dominants and subordinates, respectively. Solid and dashed lines indicate aggressive and submissive behaviors, respectively. Importantly, once dominance relationships were formed on day 1, hierarchies remained stable throughout the remainder of daily encounters. In response to the first of three social defeat encounters with a resident aggressor on day 17, the latency to submit **(D)** and duration of fighting back **(E)** were quantified. Dominants (DOM) took longer to submit than subordinates (SUB) and spent more time fighting back than SUB and dominance status controls (DSC). Letters above each bar indicate statistical significance only where different (i.e., “A” differs from “B”).

To permit territory establishment and facilitate dominance status formation, all animals in this study were individually housed for 1 week prior to social encounters. Animals assigned to dominant/subordinate groups were then weight-matched into dyads, randomly assigned as “resident” or “intruder,” and introduced daily to establish (day 1; 10 min) and maintain (days 2–8, 11–16; 5 min/day) dominance relationships (Figure 1A). Dyadic encounters were halted for stereotaxic surgery (day 9) and recovery (day 10). To index changes in agonistic behavior across time, 4 dominant and 4 submissive behaviors were scored for each animal (i.e., yes = 1, no = 0, see Figure 1B) during the early, middle, and late phases of each encounter (i.e., max scores of 3 per behavior per day). Dominance relationships formed and stabilized rapidly (Figure 1C), though one dyad was excluded for failing to do so. Dominant hamsters (*n* = 9) showed only dominant behaviors and no submissive behaviors after relationship formation on day 1; subordinates (*n* = 10) displayed the opposite pattern. Animals assigned to control groups were not paired in daily agonistic encounters and thus did not form dominance relationships. Of these controls, half received social defeat stress and are called dominance status controls (DSC, *n* = 8) and the remaining animals did not (“handled controls,” *n* = 8).

### Stereotaxic Surgery

**FIGURE 2:**
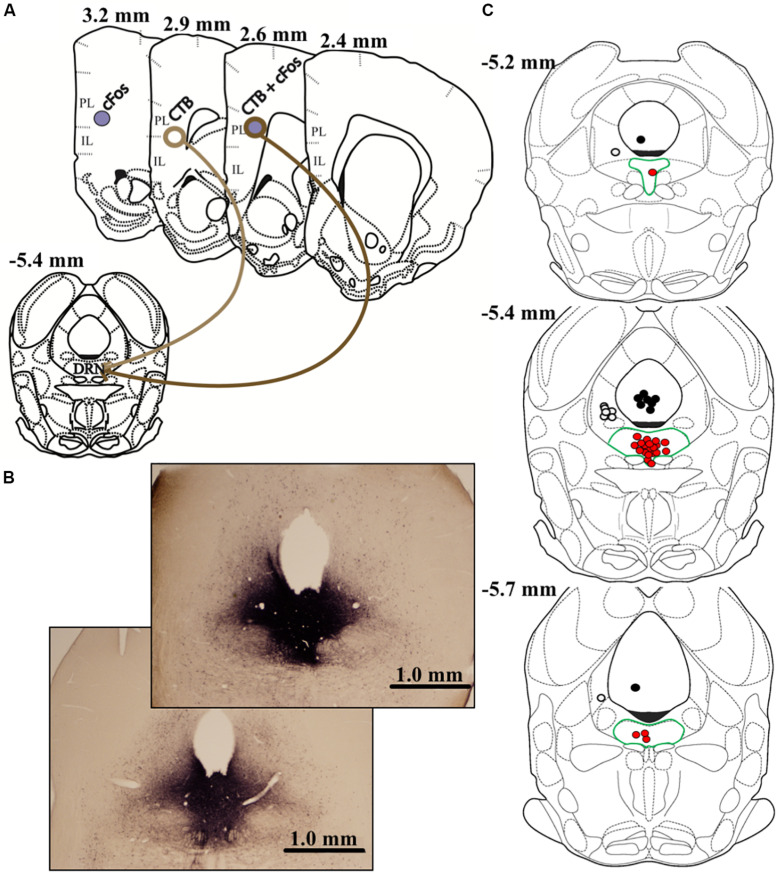
The graphic in **(A)** depicts the anatomical locations of CTB injection within dorsal raphe (DRN) and prelimbic (PL) or infralimbic (IL) subregions of vmPFC where data were quantified. Differential appearances of cFos, CTB, and cFos + CTB IR afforded by immunohistochemical procedures are also shown. cFos+ cells present as purple, solid, nuclear staining due to nickel enhancement. On the other hand, CTB+ cells appear as brown, opened staining of neuronal membranes. Dual-labeled cells, characterized by brown membranous staining around purple nuclei, indicate a DRN-projecting neuron that was activated by acute social defeat stress. Representative images of DRN injections (25 nl CTB) are shown in **(B)**. Importantly, nickel-enhanced staining was conducted to maximize contrast when verifying injection placement and CTB diffusion. Thus, CTB IR in **(B)** differs in appearance than elsewhere in this report. Notably, the top image (subordinate) represents the maximum-acceptable spread of CTB, whereas the bottom image (dominant) represents average diffusion. The graphic in **(C)** depicts all estimated injection points, some overlapping, within the DRN. Red dots depict DRN-targeted injections, and DRN is highlighted by green borders. To control for diffusions beyond DRN boundaries, injections targeting the left ventral periaqueductal gray (lvPAG) and 4th ventricle are depicted as white or black dots, respectively. Measurements on atlas templates refer to distance from bregma according to Morin and Wood (2001).

On day 9 (8 days prior to euthanasia), all animals received stereotaxic microinjections of the retrograde tracer cholera toxin B (CTB; List Biological Laboratories). Using a 20° entry angle through the left hemisphere to avoid puncturing the fourth ventricle, injections targeted the center of the DRN (5.4 μm posterior and 2.0 μm lateral to bregma, 4.6 μm below dura; Figure 41 of Morin and Wood, 2001). Following preliminary studies to optimize a pressure injection approach and minimize diffusion into neighboring areas (e.g., left ventral periaqueductal gray, lvPAG), we opted for 25-nL injections of CTB (1% in 0.9% sterile saline) across 5 min (1.25 nl every 15 s) with a 15-min diffusion period. To restrict our analyses to DRN-targeting neurons, all microinjections were verified via immunolabeling and plotted on atlas images. Importantly, we excluded all animals that did not meet the following criteria: CTB immunoreactivity (IR) filling at least 75% of DRN along both rostro-caudal and dorsal-ventral axes (*n* = 15); CTB IR extending no farther than roughly 0.5 mm beyond DRN boundaries (*n* = 17); and a central injection point deviating >0.2 mm from 5.4 mm posterior from bregma (*n* = 7). These criteria resulted in accurate injections and minimal offsite diffusion, and the center-point of successful injections generally terminated in dorsal and ventral portions of the mid-rostral DRN (Figure 2).

### Acute Social Defeat Stress

On day 17, dominant, subordinate, and DSC hamsters received acute social defeat stress as described elsewhere (e.g., Dulka et al., 2018a). Briefly, each subject was placed for 5 min into the home cage of a novel conspecific who was larger, older, sexually experienced, and prescreened for aggression (termed “resident aggressors”). This was repeated twice more with 5-min inter-trial intervals. Handled controls were exposed to three empty resident aggressor cages for the same durations. One animal was excluded due to wounding.

### Dual-Label Immunohistochemistry

To optimize our dual-label approach in Syrian hamsters, we performed preliminary studies and received advice from multiple histology experts (Alex Osmand, Robert Switzer III, and Vector Laboratories, personal communications). To minimize cross-reactivity during peroxidase steps and maximize contrast between IR of cFos, CTB, and background, we found it necessary to increase blocking efforts while reducing concentrations of antibodies and signal amplification reagents. While these modifications lowered the overall intensity of cFos IR, cFos+ puncta remained detectable at levels comparable to previous single-label studies of defeat-induced cFos IR in hamsters (e.g., Morrison et al., 2012). Nevertheless, the cFos component of our dual-label stain may underestimate total neural activity in the vmPFC, although it preserves relative differences between treatment groups.

A total of 1 h following defeat or control procedures (i.e., 90 min after defeat onset), animals were transcardially perfused with 100 mL of 0.1 M phosphate-buffered solution (PB) followed by 100 mL of 4% formaldehyde in PB (PFA). Excised brains were post-fixed (24 h; PFA) and coronally sectioned (40 μm) using a vibrating microtome (PB at 4°C) followed by free-floating IHC on every 3rd vmPFC-containing slice. Except where noted, all following steps were conducted at room temperature and separated by PB washes with 0.5% Triton X-100 (PBTx); listed solutions were based in PBTx. Briefly, following antigen retrieval (30 min; 0.5% NaBH_4_ in PB), tissue was blocked (1 h, PBTx), then incubated in CTB primary antibody [48 h; 4°C; 5% horse serum and 1:40,000 goat anti-CTB (List Biological Laboratories)] followed by a secondary antibody [1 h; 1:200 biotinylated horse, anti-goat IgG antibody (Vector Laboratories: BA-9500)]. Signal was then amplified [1 h; ABC Kit (Vector Laboratories: PK-4000)] according to kit instructions but at 1/4 the recommended dilution. Peroxidase reaction followed [10 min; PB, 0.06% H_2_O_2_, 5% 3,3′-diaminobenzidine (DAB; Sigma-Aldrich: D5905)]. Sections were then blocked [15 min; 5% Avidin-D (Vector Laboratories: SP-2001)] and incubated overnight in cFos primary antibody [1:5,000 rabbit anti-cFos (Santa Cruz: sc-52), 5% horse serum, and 5% biotin blocker (Vector Laboratories: SP-2001)]. Tissue was then incubated in secondary antibody [1 h; 1:200 biotinylated horse, anti-rabbit IgG antibody (Vector Laboratories: BA-1100)] followed by ABC (as above) and nickel-enhanced DAB solution (10 min; PB, 25% DAB, 0.3% H_2_O_2_, 0.05% H_20_N_2_NiO_14_S_2_). Tissue was then mounted following standard protocols.

### Histology

**FIGURE 3:**
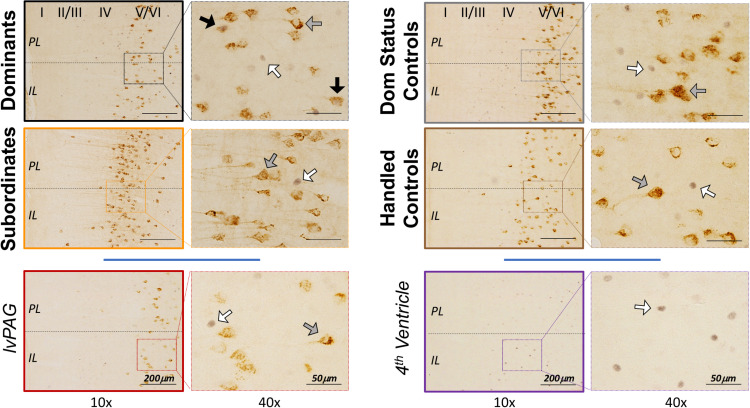
Representative vmPFC histology by treatment group, including control injections in the left ventral periaqueductal gray (lvPAG) and 4th ventricle. CTB+ cells were localized primarily in deep cortical layers (V/VI) as depicted in left panels at 10 × magnification. Arrows highlight cFos-only (white), CTB-only (gray), and cFos+ CTB cells (black) and are presented at 40 × magnification for clarity. Importantly, images here do not depict quantification or selection methods (see Histology). Notably, the dominant and subordinate subjects shown here were also selected to represent CTB diffusion in Figure 2B.

To estimate cFos, CTB, and cFos+ CTB IR in the vmPFC, cells were manually counted in tissue containing both infralimbic (IL) and prelimbic (PL) subcortical (2.4–3.2 mm anterior to bregma, approximately 6 tissue slices/animal) using an Olympus BX51 microscope and Olympus DP Controller software. Representative images are shown in Figure 3. Cortical cell layers and IL/PL boundaries were determined using nearby white matter (e.g., forceps minor of the corpus callosum), tissue contours and boundaries (e.g., rhinal fissure, lateral ventricle), somatic densities and orientations (e.g., apical dendritic branching in CTB+ cells), and grid overlays of atlas images (Figures 10–13, Morin and Wood, 2001). Although cFos IR was apparent throughout the vmPFC, CTB IR cells were localized within deep cortical layers (V/VI) with few CTB+ cells found elsewhere in vmPFC, which is consistent with anatomical studies of DRN-projecting cells in other rodents (Vertes, 2004). For this reason, we focused on layers V/VI following a protocol described elsewhere (Dulka et al., 2018a). Briefly, we manually counted all IR cells that were visible at 20 × magnification within the digitized viewfinder (i.e., “image box,” 439 μm × 330 μm). Data are presented as summed cell counts across two stacked image boxes, thus 439 μm × 660 μm × 40 μm (width × height × thickness), per subregion per hemisphere per subregion. Care was taken to avoid placing image boxes at the IL/PL junction. Single- and dual-labeled cells were confirmed on a cell-by-cell basis at 40 × magnification using lighting adjustments and progressive focus through the tissue to distinguish background and overlapping, single-labeled cells. Reliability (>90% agreement) was established across multiple blinded experimenters.

### Statistical Analyses

Parametric data were analyzed using a paired Student’s *t*-test or analysis of variance (ANOVA) followed by Tukey’s *post hoc* tests, where appropriate. Non-parametric data were analyzed using Kruskal–Wallis tests followed by Dunn’s *post hoc* tests, and Spearman’s rank correlations were used to assess the association between histological results and behavior during social defeat. For all immunohistochemical analyses, no differences were found along rostro-caudal axes or across hemispheres, so data were pooled accordingly. Statistical significance was set at *p* < 0.05. All graphical data are reported as mean ± SEM and textual data for behavioral analyses are reported as pooled mean ± SD except where differing statistically from other groups. Histological data are presented in Table 1 as mean ± SD or Spearman’s r, where appropriate.

**TABLE 1:** Means ± standard deviations of cell counts across histological categories are organized by treatment condition.

	**cFos only**		**CTB only**		**Dual (cFos+ CTB)**		**Dual (as% CTB)**		**Dual (as% cFos)**	
**Treatment (*n* = 8–10/group)**	**PL**	**IL**	**PL**	**IL**	**PL**	**IL**	**PL**	**IL**	**PL**	**IL**
Dominants	27.2* ± 8.6	19.9* ± 7.6	45.0 ± 10.3	46.9 ± 12.1	2.1*# ± 1.3	2.2*# ± 1.3	4.7*# ± 2.2	4.4*# ± 2.2	7.9*# ± 4.3	12.3*# ± 7.5
Subordinates	**9.9# ± 7.3**	**9.3# ± 5.5**	52.5 ± 16.9	51.5 ± 13.1	0.1 ± 0.2	0.2 ± 0.3	0.3 ± 0.3	0.4 ± 0.5	2.1 ± 3.2	2.2 ± 2.9
Dom. status controls	28.2* ± 8.6	21.0* ± 6.5	56.9 ± 13.7	47.7 ± 16.3	0.7 ± 0.7	0.5 ± 0.3	1.1 ± 1.0	1.1 ± 0.7	2.5 ± 2.1	2.2 ± 1.0
Handled controls	**8.7# ± 4.9**	**7.2# ± 3.4**	45.9 ± 15.4	45.0 ± 13.6	0.4 ± 0.5	0.2 ± 0.2	0.3 ± 0.4	0.4 ± 0.6	1.5 ± 2.3	4.2 ± 6.8
*lvPAG injection*	*11.8* ± *4.2*	*10.4* ± *4.8*	*21.4* ± *10.4*	*17.7* ± *7.5*	*n/a*	*n/a*	*n/a*	*n/a*	*n/a*	*n/a*
***Spearman’s r (n*=*35):***										
Fighting back	**0.38†**	0.26	−0.03	0.06	**0.68‡**	**0.76‡**	**0.67‡**	**0.78‡**	**0.61‡**	**0.70‡**
Latency to submit	0.34	**0.43†**	−0.25	−0.14	0.299	**0.53†**	0.33	**0.53†**	0.15	0.38

Spearman’s correlation coefficients at the bottom of the table refer to correlations between cell counts and proactive agonistic behaviors during the first of three acute defeat episodes. lvPAG denotes control injections of 4 dominants and 4 subordinates that targeted the left ventral aspect of the periaqueductal gray only and lvPAG cell counts were not included in ANOVA. Ventricle only injections yielded no CTB IR cells and are not shown. Significance is indicated as follows: bold lettering denotes statistical significance where *p* < 0.05. For histological comparisons: asterisks (*) denote significant difference of cell counts when compared with subordinates; pound signs (#) denote significant difference when compared with dominance status controls. For Spearman’s correlations: daggers denote significant correlations where † = *p* < 0.05; double daggers denote significant correlations where ‡ = *p* < 0.001. Sample sizes by group: Dominants, *n* = 9; Subordinates, n = 10; and for Dominant status controls, Handled controls, lvPAG injection, and Ventricle injection, n = 8/group.

## Results

### Behavioral Data

All dyads formed stable dominance relationships on day 1 (Figure 1C). During social defeat encounters, there were no differences between treatment groups in received duration of aggression (95.70 ± 37.27, *F*_2_,_24_ = 0.3236, *p* = 0.7266) or total attacks (9.85 ± 5.10, *F*_2_,_24_ = 0.3273, *p* = 0.7240). However, 8 of 9 dominants, 2 of 8 DSC, and 0 of 10 subordinates fought back against the resident aggressor in the first defeat encounter. Kruskal–Wallis tests revealed group differences in latency to submit [χ^2^(2) = 8.910, *p* = 0.0116, Figure 1D] and duration of fighting back [χ^2^(2) = 14.58, *p* = 0.0007, Figure 1E]. *Post hoc* analyses revealed that dominant animals took longer to submit (62.67 ± 81.87) and fought back for a longer duration (26.67 ± 42.07) than subordinates (3.89 ± 3.22, 0 ± 0, respectively) yet differed from DSC only in duration of fighting back (3.88 ± 9.80, latency to submit = 40.63 ± 46.20). These results are consistent with previous studies using this model.

### Immunohistochemistry

#### Localization of CTB Injections Into DRN

Following exclusions (described above), most injections were confined to and filled the DRN (*n* = 5–7/group). However, CTB IR was occasionally detected beyond DRN boundaries (*n* = 1–4/group), with the majority diffusing along the needle tract in lvPAG or, potentially, into the fourth ventricle.

#### Quantification of vmPFC Cells

The means and SDs for CTB, cFos, and CTB+ cFos IR cell counts from deep cortical layers of PL and IL cortices of each group are presented in Table 1. Dominants, subordinates, DSC, and handled controls did not differ in CTB+ cells in PL (*F*_3_,_31_ = 1.301, *p* = 0.2910) or IL (*F*_3_,_31_ = 0.365, *p* = 0.7785). Subsequently, a paired Student’s *t* test of pooled cell counts revealed no significant difference between PL and IL subregions (*t* = 1.364, df = 34, *p* = 0.18).

**FIGURE 4:**
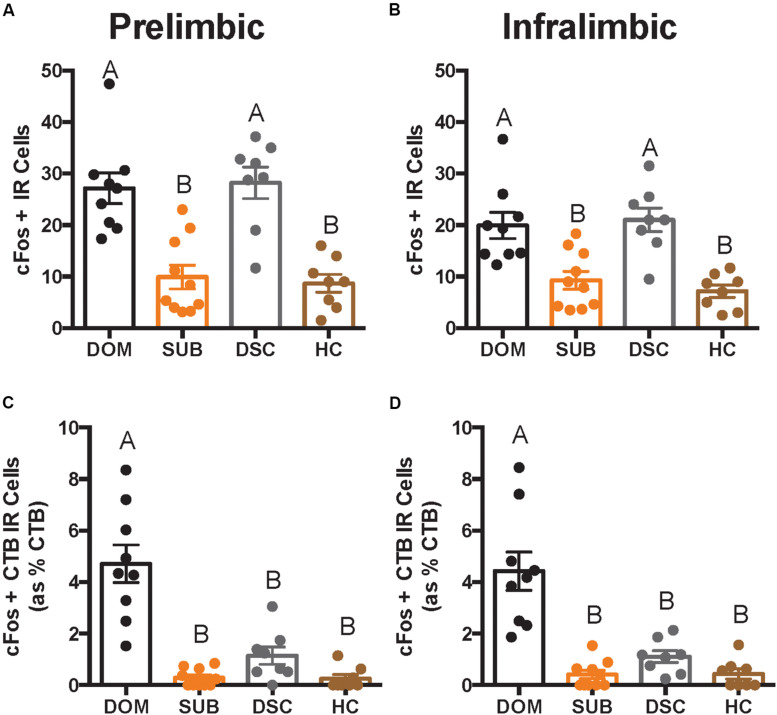
Total number of cFos IR cells and cFos+ CTB IR cells in deep cortical layers is shown as quantified across an average of 12 replicates within a volume of 439 μm × 660 μm × 40 μm (width × height × thickness) in a *per subregion*, *per hemisphere*, *per slice* manner. In both prelimbic **(A)** and infralimbic **(B)** subregions, dominant hamsters (DOM) differed significantly in total cFos IR when compared with subordinate (SUB) and handled controls (HC), but not dominance status controls (DSC). On the other hand, DOM differed from all other groups in dual-labeled cells (shown as a percentage of total CTB+ cells) in both prelimbic **(C)** and infralimbic **(D)** cortices. Letters above each bar indicate statistical significance only where different (i.e., “A” differs from “B”).

There were significant differences in total cFos+ cells of the PL (*F*_3_,_31_ = 16.66, *p* < 0.0001; Figure 4A) and IL (*F*_3_,_31_ = 12.08, *p* < 0.0001; Figure 4B). *Post hoc* analyses reveal that dominants and DSC displayed more PL and IL cFos+ cells, respectively, compared to subordinates and handled controls, but did not differ from each other in either subregion.

Colocalization of CTB and cFos also differed in the PL (*F*_3_,_31_ = 26.22, *p* < 0.0001; Figure 4C) and IL (*F*_3_,_31_ = 21.34, *p* < 0.0001; Figure 4D). *Post hoc* analyses revealed that dominant animals alone showed more CTB+ cFos IR cells compared to all other groups in both PL and IL.

To test whether vmPFC activity was associated with behavioral responses during social defeat, Spearman correlation coefficients (summarized in Table 1) were determined between cell counts and proactive behaviors during the first defeat episode. Most notably, strong relationships were detected for the duration of fighting back and the number of CTB+ cFos IR cells in the PL (*r* = 0.68; *p* < 0.0001) and IL (*r* = 0.78; *p* < 0.0001).

#### Off-Site Injections Into the Left Ventral Periaqueductal Gray (lvPAG) and Fourth Ventricle

We performed additional analyses to account for diffusion of CTB into the lvPAG and/or fourth ventricle. First, all animals with any CTB IR in lvPAG were removed from analyses and statistics were recalculated. Importantly, CTB+ cFos IR results changed minimally in PL (*F*_3_,_23_ = 27.52, *p* < 0.0001) and IL (*F*_3_,_23_ = 19.04, *p* < 0.0001) and dominants remained significantly different from others. Second, we performed additional surgeries (*n* = 8/region; 4 dominant, 4 subordinate) where injection locations targeted the lvPAG (5.275 μm below dura) or fourth ventricle (1.6 μm lateral from bregma; 4.4 μm below dura). Animals with lvPAG injections had fewer CTB+ cells in the IL and PL compared to DRN-injected animals, and no double-labeled cells were detected in the vmPFC (see Table 1 and Figure 3). In fourth ventricle-injected animals, there were no detectable CTB+ cells within vmPFC and no further analyses were conducted.

## Discussion

vmPFC-DRN projections are comprised primarily of glutamatergic efferents that preferentially synapse onto GABAergic interneurons within the lateral wings of the DRN, which then synapse onto and inhibit serotonin-producing cells along the DRN’s midline (e.g., Jankowski and Sesack, 2004; Challis et al., 2014). Given that acute stress-induced activation of serotonergic cells is positively correlated with stress susceptibility (Amat et al., 2005; Cooper et al., 2008, 2017; Paul et al., 2011), activation of vmPFC-DRN projections is thought to functionally suppress the DRN’s response during acute stress and buffer its negative consequences. Indeed, vmPFC-DRN cell recruitment is associated with indices of resilience to non-social laboratory stressors such as tail shock (Baratta et al., 2009, 2019), elevated plus maze (Faye et al., 2020), and forced swim (Warden et al., 2012). In sum, selectively engaging the vmPFC-DRN circuit appears to promote resistance to the effects of acute stress, perhaps by gating proactive behavioral coping strategies. Accordingly, the vmPFC-DRN pathway has recently been dubbed “the hope circuit” (Maier and Seligman, 2016).

In this study, we demonstrate that experiencing social dominance for 14 days in male Syrian hamsters selectively activates vmPFC-DRN-projecting neurons during an acute social defeat stressor. Furthermore, greater defeat-induced activation of the vmPFC-DRN pathway is directly associated with longer latencies to submit to an aggressor as well as increased likelihoods and durations of fighting back, which are proactive behavioral strategies thought to promote stress resilience. For example, counterattacking aggressors is linked to less disruption of circadian heart rate and body temperature rhythms (Meerlo et al., 1999). Similarly, actively resisting an opponent and delaying the onset of social defeat are associated with reduced hypothalamic–pituitary–adrenal (HPA) axis activity (Veenema et al., 2004; Walker et al., 2009), more rapid HPA axis habituation (Wood et al., 2010), and decreased corticotropin-releasing hormone mRNA in the PFC and hippocampus (McQuaid et al., 2013). Behaviorally, resisting defeat coincides with decreased immobility in a forced swim test (Veenema et al., 2005; Wood et al., 2010), increased active avoidance in a shuttle box (Benus et al., 1989), active shock probe burial (Koolhaas et al., 2010), and reduced conditioned fear of the defeat context (Walker et al., 2008). Similarly, counterattacking aggressors during social defeat stress is associated with a reduced conditioned defeat response, marked by avoidance of and submission to a non-threatening social stimulus (Morrison et al., 2014; Dulka et al., 2018b).

Our results also support the hypothesis that stress resilience can be learned and is associated with experience-dependent vmPFC plasticity. In the current study, we found that only dominants showed vmPFC-DRN activity above the levels displayed by non-defeated, handled controls, suggesting that maintaining social dominance leads to neural plasticity that allows recruitment of vmPFC-DRN neurons during stress. This is consistent with our previous reports that proactive behaviors during defeat, increased cFos expression in vmPFC, and reduced conditioned defeat develop gradually as dominance is maintained for 2 weeks (Morrison et al., 2014). These status-dependent effects are likely mediated by vmPFC since pharmacological inhibition of the vmPFC during social defeat prevents resistance to conditioned defeat in dominant hamsters only (Morrison et al., 2013). Dominants also display less neural activity in the DRN compared to subordinates during both social defeat and physical restraint stress (Cooper et al., 2017), which is important given that DRN activity is necessary to both acquire and express a conditioned defeat response (Cooper et al., 2008). Together, this suggests that vmPFC-DRN circuit plasticity results from learning social dominance in hamsters. Such an interpretation is consistent with the learned controllability model studied extensively by Maier and colleagues (Maier and Seligman, 2016). Briefly, learning to control a stressor’s duration by proactively turning a wheel to terminate a tail shock promotes immediate resistance to learned helplessness and produces *behavioral immunization* by buffering responses to future, even uncontrollable stressors. The resilience afforded by learning controllability is dependent on vmPFC (Amat et al., 2008) and dorsal striatum (Amat et al., 2014), coincides with increased vmPFC-DRN activity (Baratta et al., 2009, 2019), and is associated with decreased raphe activity (Amat et al., 2005, 2014). With this said, more work is needed to elucidate vmPFC-DRN recruitment mechanisms in dominant hamsters, including whether they similarly engage the extended networks seen in stressor controllability by learning social dominance. Furthermore, a causal role for this circuit in promoting stress resistance will need to be determined.

While the contributions of vmPFC-DRN neurons to learning (i.e., during dominance relationships) or conferring (i.e., during social defeat stress) stress resilience can be addressed by viral-mediated, circuit-manipulation approaches, such designs have produced equivocal results. For example, Berton and colleagues (Challis et al., 2014) found that optogenetically driving vmPFC-DRN neurons induced electrophysiologic changes in DRN-GABA neurons that appear capable of producing serotonergic hypofunction, which is characteristic of MDD in humans. Berton’s group also found that repeated vmPFC-DRN photostimulation for 10 days in non-stressed mice produced a social avoidance response that resembles susceptibility to chronic social defeat stress (CSDS). Finally, photoinhibition of vmPFC-DRN terminals after daily CSDS physical defeats reduced socially avoidant behaviors. The authors thus concluded that vmPFC-DRN activity biases negative socioemotional valence and drives avoidance via serotonergic dysfunction. Importantly, these findings are inconsistent with the implications of the current study as well as the possibility that maintaining social dominance repeatedly activates vmPFC-DRN neurons on a daily basis. Intriguingly, Berton’s group has also shown that deep brain stimulation of vmPFC, and thus broader network engagement, normalizes both serotonergic disruptions and social avoidance in CSDS-susceptible mice (Veerakumar et al., 2014). Similarly, broad optogenetic, burst-like stimulation of vmPFC reduced CSDS-induced social avoidance (Covington et al., 2010). However, when stimulation was restricted to cortical layer V, this effect on CSDS-induced social avoidance was lost, though a reduction of other anxiety-like behaviors was gained (Kumar et al., 2013). In contrast, Warden et al. (2012) showed that broad optogenetic vmPFC stimulation did not, yet stimulating vmPFC terminals within DRN did, promote escape-oriented forced swimming behaviors.

Expounding our results while integrating such disparate literature encourages consideration of how vmPFC-DRN neurons are recruited, what and where their terminals innervate, and whether a particular artificial activation/inhibition method has appropriately recapitulated endogenous engagement. Given that our dual-label approach identified only one IEG, it is highly unlikely that our study reflects all vmPFC-DRN cells activated by acute stress in dominant hamsters. Still, it is noteworthy that both we and Baratta et al. (2009), who utilized a similar approach, identified relatively small percentages of DRN-projecting neurons as activated by acute stress in resilient animals. Further, the specificity of vmPFC-terminal innervation during social defeat in dominant hamsters is currently unknown, though it is likely that GABAergic synapses in lateral DRN are not the only recipients of top-down regulation, and vmPFC-stimulated serotonin release is possible. Indeed, status-dependent, acute stress-induced cFos profiles within DRN differ in topographical and cell-specific manners (Cooper et al., 2009, 2017). Relevantly, both serotonergic and non-serotonergic DRN populations residing within vmPFC-terminal fields are functionally heterogeneous (Lowry, 2002; Marinelli et al., 2004; Calizo et al., 2011). For example, serotonergic cells in dorsal and caudal DRN, an area with dense vmPFC innervation (Challis et al., 2014), can facilitate anxiety and aversion via projections to the basolateral amygdala (Hale et al., 2012; Paul et al., 2014). On the other hand, vmPFC-mediated activation of serotonergic cells within the dorsal and lateral DRN leads to inhibition of dorsal PAG (dPAG) activity and suppression of panic-like escape behavior (Yamashita et al., 2017). Thus, a vmPFC-DRN-dPAG circuit could promote proactive behaviors, such as fighting back or resisting defeat, via the serotonin-dependent, *panic inhibition system* (Paul et al., 2014). Anxiety suppression has also been implicated via vmPFC-induced, serotonin-dependent feedback in the forebrain. Injecting a 5-HT4 receptor agonist into the vmPFC reduced anxiety-like behaviors, as did optogenetic stimulation of vmPFC terminals; however, photoinhibition abolished this effect (Faye et al., 2020). Altogether, these studies highlight the functional heterogeneity of DRN cells as well as the vmPFC-DRN circuit as a whole and support the notion that select vmPFC-DRN ensembles may differentially contribute to stress-induced behavior. Taken together, artificially recruiting vmPFC-DRN cells with minimal selectivity for specific vmPFC afferents may not adequately reflect endogenous activity or function. Inasmuch, broadly manipulating this pathway could produce supraphysiologic circuit activity that overemphasizes or misrepresents its function and could also produce compensatory mechanisms that overshadow the roles of specific neuronal ensembles.

In summary, the vmPFC-DRN circuit has demonstrable roles in promoting proactive behaviors during acute trauma and activity of this pathway has been associated with resilience in multiple animal models. Our results are not only consistent with such reports, but these findings extend to the social domain and implicate a subset of vmPFC-DRN projections in experience-dependent resistance to social stress. What lies upstream and downstream of vmPFC-DRN neurons during dominance maintenance is currently unknown, and thus more work is required. Moreover, elucidating endogenous recruitment mechanisms and terminal field innervation properties of vmPFC-DRN afferents may ease efforts to disambiguate a causal role for vmPFC-DRN activation in social stress resilience and top-down control of the serotonergic system.

## Data Availability Statement

The datasets generated for this study are available on request to the corresponding authors.

## Ethics Statement

This study was reviewed and approved by The Institutional Animal Care and Use Committee at The University of Tennessee, Knoxville and all procedures were conducted in line with guidance from The National Institutes of Health.

## Author Contributions

AG and MC designed the experiments. AG led the experiments. TC, NG, and BD assisted in conducting experiments. AG analyzed the data and wrote the manuscript. All authors edited and reviewed the manuscript for publication.

## Conflict of Interest

The authors declare that the research was conducted in the absence of any commercial or financial relationships that could be construed as a potential conflict of interest.
